# Dupilumab's Impact on Blood Parameters in Nasal Polyposis: 18-Month Follow-Up in Real Life

**DOI:** 10.1155/2023/4027701

**Published:** 2023-09-15

**Authors:** Antonella Loperfido, Andrea Ciofalo, Carlo Cavaliere, Elona Begvarfaj, Francesca Cascone, Giacomo Alfonzo, Rosalba Cadeddu, Stefano Millarelli, Gianluca Bellocchi, Antonio Greco, Marco de Vincentiis, Simonetta Masieri

**Affiliations:** ^1^Otolaryngology Unit, San Camillo Forlanini Hospital, Rome, Italy; ^2^Department of Sense Organs, Sapienza University, Rome, Italy; ^3^Department of Oral and Maxillofacial Sciences, Sapienza University, Rome, Italy

## Abstract

**Background:**

Dupilumab represents the first approved biological for severe uncontrolled chronic rhinosinusitis with nasal polyps (CRSwNP).

**Objective:**

Aim of this paper is to provide a multicentric real-life study about treatment with dupilumab for CRSwNP with a special focus on blood parameters and IgE, IgG, and IgA.

**Method:**

A retrospective data collection was jointly conducted at the Otolaryngology departments of San Camillo Forlanini Hospital and University of Rome “La Sapienza” from December 2020 to January 2023.

**Results:**

A total of 130 patients were included in the study. Monitoring our patients for 18 months, we observed a reduction in nasal polyposis and an improvement in symptoms and their impact on quality of life. Regarding blood tests, a transient increase in blood eosinophils was found in most cases. Total IgE showed a gradual decrease in values. IgG and IgA also showed a slight reduction of values, while remaining within normal ranges.

**Conclusion:**

To the best of our knowledge, this is the first study to evaluate the impact of dupilumab on several blood parameters in patients receiving treatment for CRswNP. Further studies are needed to confirm our results and to understand the underlying immunological mechanisms.

## 1. Introduction

Chronic rhinosinusitis with nasal polyps (CRSwNP) represents a chronic inflammatory disorder of the nasal mucosa and paranasal sinuses [1–3].

Biological treatments have recently changed the therapeutic paradigm of several chronic eosinophilic diseases, especially asthma [4, 5] and atopic dermatitis (AD) [6–8], by targeting specific inflammatory mediators. These molecules have also proved to be effective in severe uncontrolled CRSwNP; therefore, this topic is rapidly gaining particular interest among clinicians and researchers in this field [2, 9–11].

Monoclonal antibodies (Mabs) may act on type 2 inflammatory response in several ways: by targeting the IL-5 pathways (mepolizumab or benralizumab) [12–14], neutralizing the IgE-mediated response (omalizumab) [15], and acting against IL-4 and IL-13 signaling (dupilumab) [16, 17], demonstrating, the three drugs mentioned, efficacy in the treatment of CRSwNP [18–22].

In particular, dupilumab is a fully human Mab that inhibits both cytokines IL-4 and IL-13 and has currently become a cornerstone in the treatment strategy for several type 2 inflammation-related diseases, such as asthma and AD [23]. Dupilumab is the first biological treatment approved by Agenzia Italiana del Farmaco (AIFA) on December 2020 for adult patients with severe CRSwNP in addition to topical treatment with intranasal corticosteroids (INCS), in those cases which are uncontrolled with oral steroids and/or surgery [24].

Recent data from clinical practice support using dupilumab as a valid treatment option for CRSwNP forms that are unresponsive to conventional therapies, with good results in disease control, reduced need for systemic corticosteroids (SCS) and sinonasal surgery, improved quality of life, and olfactory recovery [25–28]. Some studies about dupilumab in AD describe its impact in terms of routine blood parameters [29, 30]. However, the only effect described so far regards eosinophil count, reporting the possibility of hypereosinophilia which, however, is typically transient in most cases [31, 32].

The aim of this paper is to describe a multicenter real-life study about dupilumab in the management of uncontrolled severe CRSwNP with a special focus on blood parameters, including eosinophils, neutrophils, lymphocytes, basophils, IgE, IgG, and IgA.

## 2. Methods

In January 2023, a retrospective data collection was jointly conducted at the Otolaryngology-Head and Neck Surgery departments of San Camillo Forlanini Hospital and the University of Rome “La Sapienza.” Both centers shared data on patients with uncontrolled severe CRSwNP treated with dupilumab starting December 2020.

Ethics committee approval was obtained (Prot. N 411/CE Lazio1 19 Apr 2022), and informed consent on privacy and use of clinical data was obtained from patients at the time of collection.

The AIFA treatment plan for dupilumab requires a minimum age of 18 years, diagnosis of CRSwNP confirmed by nasal endoscopy, severe stage of the disease as assessed by nasal polyp score (NPS) ≥5 or Sinonasal Outcome Test-22 items (SNOT-22) ≥50, failure or refusal of previous corticosteroid, and/or surgical treatment [33]. Exclusion criteria for starting treatment were pregnancy, patients who refused to start the biological treatment, radiochemotherapy for cancer in the last 12 months, and patients who have not signed the consent to the use of their data.

Patients were evaluated at baseline before starting dupilumab (time 0 or T0), at 6 months (T1), at 12 months (T2), and at 18 months (T3) from the first administration.

Before starting dupilumab, each patient was systematically assessed to obtain a comprehensive anamnestic collection, including sex, age, concomitant allergies, asthma concurrence, comorbid gastroesophageal reflux disease (GERD), and nonsteroidal anti-inflammatory drugs (NSAIDs) intolerance. A detailed anamnestic collection of any past surgical procedures for CRSwNP before starting dupilumab was also performed. Moreover, at baseline, a complete blood count, including eosinophils, neutrophils, lymphocytes, basophils, and immunoglobulin (Ig)E, IgG, and IgA assays, was obtained for each patient.

Another systematic investigation performed was nasal endoscopy to objectively assess the presence and extent of nasal polyposis and, therefore, quantify the severity of the disease through NPS [34]. The assessment of the quality of life (QoL) was carried out through the visual analog scale (VAS) and the SNOT-22 [35]. VAS evaluates the intensity of specific symptoms, measured with a scale of values ranging from 0 to 10, while the SNOT-22 is a validated disease-specific score that presents a minimal clinical important difference (MCID) and normative values [36, 37]. Evaluated symptoms included nasal obstruction, nasal secretion, loss of smell, postnasal drip, and headache [38]. We performed the Sniffin' Sticks-16 Identification Test (SSIT-16) to evaluate the olfaction [39].

Then, during all follow-up visits, patients underwent nasal endoscopic evaluation, QoL assessment through SNOT-22 and VAS tests, SSIT-16 for the olfaction, and, finally, blood tests.

The statistical analysis was performed by the software Statistica 12 (StatSoft). We used analysis of variance (ANOVA) for repeated measures and Newman–Keuls test as post hoc. Values are reported as a mean (range), mean (SD), or percentage of the total. A value of *p* < 0.05 was considered to be statistically significant.

## 3. Results

A total of 130 patients were included, whose 77 patients were males (59.3%) and 53 were females (40.7%), showing a slight male prevalence (F : M = 1 : 1.4). The mean age was 56.8 years (20–90 years). The mean body mass index (BMI) was equal to 24.5 (19.1–38.7), demonstrating a normal average weight in the cohort [40].

Among our patients, 30.7% were smokers, and 54.5% suffered from concomitant asthma. In 66.9% of cases, we found evidence of concomitant allergies, especially for dust mites (*Dermatophagoides farinae* and *Dermatophagoides pteronyssinus*), grasses, and Parietaria; 14.6% of patients suffered from NSAIDs intolerance, and 22% reported GERD as comorbidity. In our series, 83.2% of patients underwent at least one surgery before starting dupilumab: in 42.4% of cases, it was only a single functional endoscopic sinus surgery (FESS) procedure before starting biologic therapy, whereas in 40.8% of cases, dupilumab was the therapeutic choice after two or more FESS procedures. The range of the number of surgeries performed before the biological therapy was from 1 to 12. A summary of all described anamnestic patients' features can be found in Table 1.

During the follow-up, we could verify a significant improvement in NPS, SNOT-22, VAS, and olfaction.

**Figure 1:**
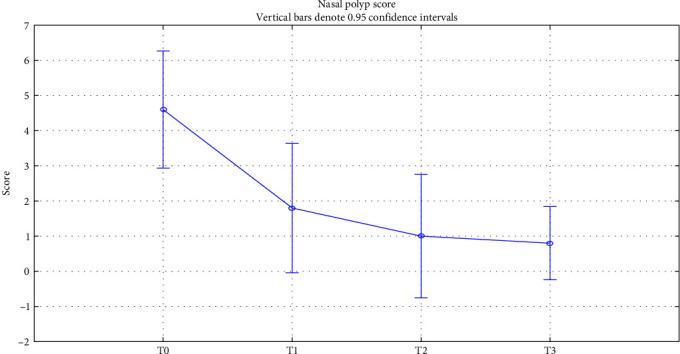
Nasal polyp score. Nasal polyp score (NPS) change over time. T0: baseline; T1 : 6 months of treatment (*p*=0.001); T2 : 12 months of treatment (*p* < 0.001); T3 : 18 months of treatment (*p* < 0.001).

Concerning the nasal polyps, evaluated through a periodically performed nasal endoscopy, we found a gradual improvement of NPS. The mean value before starting dupilumab was 4.7 (1.7) and significantly decreased to 0.4 (0.6) after 1.5 years (*p* < 0.001). After 6 months of therapy, the score significantly dropped to 1.6 (1.5) (*p*=0.001), and after 12 months, we observed a value of 0.9 (1.1) (*p* < 0.001). The improvement of the mean NPS value is shown in Figure 1.

Dupilumab also significantly impacted QoL improvement, as demonstrated by the trend of SNOT-22 and VAS. Concerning SNOT-22, the mean value before starting dupilumab was 51.6 (20.2). After 6 months, it reached the value of 21.8 (15.6) (*p* < 0.001). After 12 months, the value continued to decrease to 17.7 (14.0) (*p* < 0.001), and at 18 months, the recorded value was 14.8 (12.3) (*p* < 0.001).

Regarding the investigated symptoms through VAS, the mean value at baseline was 35.4 (8.8) and significantly decreased to 8.2 (6.9) (*p* < 0.001) at 18 months. After 6 months, it was 13.8 (9.1) (*p* < 0.001). After 12 months, the value was 9.9 (8.4) (*p* < 0.001).

Concerning the olfaction, before starting dupilumab, 62.6% of patients were anosmic to the SSIT-16, 23.7% were hyposmic, and 13.7% had normal olfaction. After 6 months, anosmic patients decreased to 11.4%, hyposmic patients were 24.5%, and 64.1% were normosmic. After 1 year of treatment, only 6.9% of all patients were anosmic, 27.6% of patients were hyposmic, and 65.5% of all cases were normosmic. Finally, after 18 months of biological therapy, nobody was anosmic; only 6.7% of all patients were hyposmic, and 93.3% of all cases were normosmic. No patient reported dysosmia or hyperosmia during follow-up.

Concerning the safety profile of dupilumab, 32 patients (24.6%) showed side effects, mostly mild and transient. These mainly included joint pain, redness, swelling, irritation and/or pain at the injection site, headache, asthenia, and eye dryness. In five patients, however, dupilumab had to be discontinued. One patient manifested diffuse skin rash and pruritus on the upper and lower limbs, unresponsive to the antihistamine. He then performed a dermatologic evaluation, which diagnosed irritative dermatitis. Blood exams showed an increase in eosinophilia (0.80 cell × 10^9^/L). After 2 months, the patient discontinued the biologic due to the persistence of rash and pruritus and despite the local and systemic therapy prescribed by the dermatologist. One further patient manifested skin effects, particularly the onset of guttate psoriasis in the fourth month of therapy. The control blood count showed no increase in eosinophils, and the value, slightly above the limits, was essentially the same as at baseline (0.7 cell × 10^9^/L). Again, the patient was referred for dermatologic evaluation, and because the disease persisted despite therapy, the biologic was discontinued. Finally, three patients stopped the medication for severe joint pain, two patients suffered from arm joint pain (elbow and wrist) unrelated to the drug injection site, and one patient from knee joint pain. All three patients underwent a rheumatological examination; the values of blood eosinophils were 0.9, 1.0, and 1.2 cell × 10^9^/L, and the antineutrophil cytoplasmic antibodies (ANCA) tests were negative. In agreement with the rheumatologist, it was decided to interrupt the therapy with the monoclonal antibody. The three patients reported an improvement in symptoms 4 weeks after discontinuing the drug.

Regarding blood tests, lymphocytes, neutrophils, and basophils remained within the normal range.

**Figure 2:**
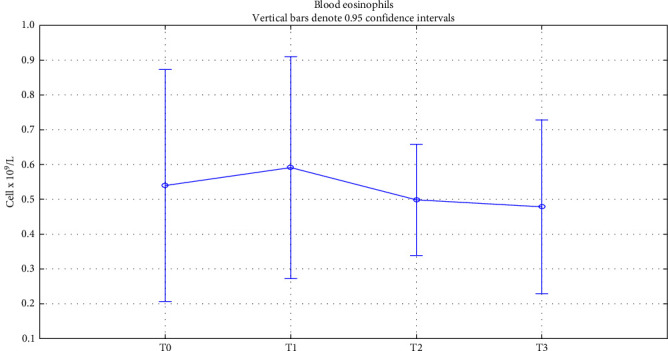
Eosinophils. Eosinophils change over time. T0: baseline; T1 : 6 months of treatment; T2 : 18 months of treatment; T3 : 18 months of treatment.

A transient increase in blood eosinophils was found in most cases; however, only in seven patients out of 130 (5.38% of all cases), eosinophils were >1.5 × 10^9^/L, thus being consistent with a condition of hypereosinophilia. Rapid and spontaneous resolution occurred in most cases without requiring any steroid treatment or dupilumab discontinuation, as proposed in the recent literature [41].  Figure 2 reports the average eosinophil trend.

**Figure 3:**
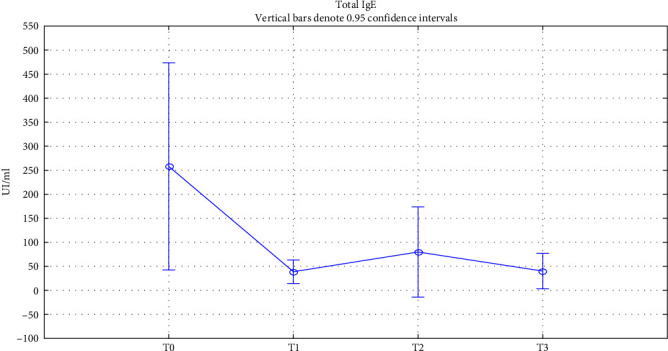
Total serum IgE trend. Total serum IgE change over time. T0: baseline; T1 : 6 months of treatment; T2 : 18 months of treatment; T3 : 18 months of treatment.

**Figure 4:**
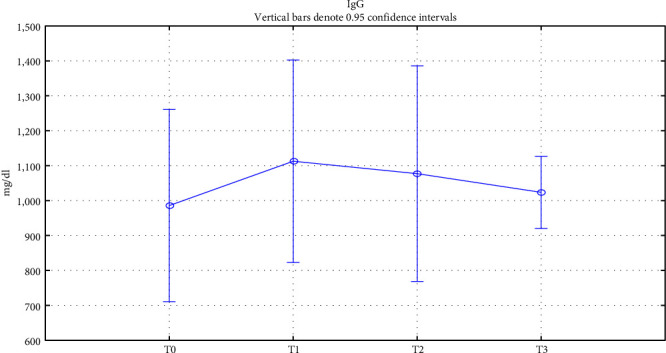
Total serum IgG trend. Total serum IgG change over time. T0: baseline; T1 : 6 months of treatment; T2 : 18 months of treatment; T3 : 18 months of treatment.

**Figure 5:**
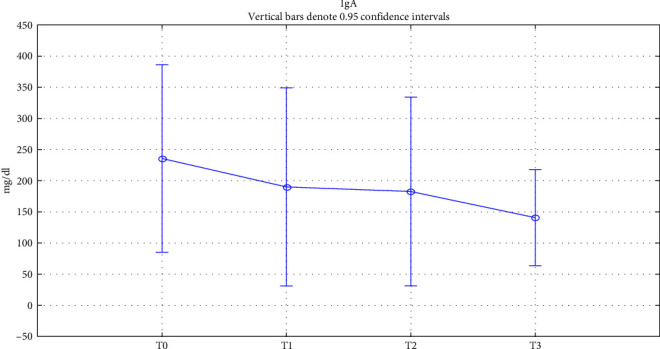
Total serum IgA trend. Total serum IgA change over time. T0: baseline; T1 : 6 months of treatment; T2 : 18 months of treatment; T3 : 18 months of treatment.

Finally, to assess patient immunity, we studied the trend of total IgE, IgG, and IgA in serum during treatment. Total IgE showed a significant gradual decrease in values from the baseline value until 18 months of treatment (*p* < 0.05) (Figure 3). IgG and IgA also showed a nonsignificative variation in values while remaining within normal ranges, as reported respectively in Figures 4 and 5. All data relating to changes from baseline values of the investigated factors are summarized in Table 2.

## 4. Discussion

In Western countries, approximately 80% of diffuse CRS are characterized by a type 2 inflammatory response driven by activation of type 2 CD4+ helper cells and innate lymphoid type 2 cells, resulting in the production of proinflammatory cytokines (IL-4, IL-5, IL-13) and tissue infiltration of inflammatory cells as eosinophils, mast cells, and basophils [42–45]. Specifically, eosinophilia represents the typical feature of type 2 inflammation, leading to more severe symptoms, a high rate of recurrences, and a higher prevalence and severity of concomitant asthma [46, 47]. In fact, it has been widely demonstrated that type 2 inflammation is the dominant driver of several chronic inflammatory conditions such as asthma, CRSwNP, AD, and eosinophilic esophagitis [48]. In particular, there is a solid epidemiologic, pathogenetic, and clinical association between CRS and asthma, leading to the global concept of unified airway disease (UAD) [49, 50]. According to the UAD concept, upper and lower airways form a single functional unit, with upper and lower airway diseases frequently co-occurring, specifically CRSwNP and asthma [51]. This pathological condition causes a high impact on the health-related quality of life and productivity of patients, with frequent recurrence despite pharmacological therapy with corticosteroids and/or surgical treatments [52].

Treatment guidelines for CRSwNP recommend a stepwise approach based on disease severity, including nasal irrigation with saline, topical/local INCS, and short courses of systemic corticosteroids (SCS) for more severe forms of the disease. In drug-refractory cases, therapeutic management is endoscopic sinus surgery (ESS). However, postoperative recurrence of nasal polyps is common, with reported recurrence rates of approximately 40% of patients within 18 months of ESS and nearly 80% within 12 years [53, 54]. Nevertheless, it should be noted that meta-analysis demonstrates that the percentages of revision surgery are much lower than the recurrence rate, attesting between 14% and 24% and recognizing asthma and NSAIDs intolerance as the main risk factors [55]; it has also been observed that the recurrence percentages appear to be lower in patients treated with more complex surgery [56, 57].

Furthermore, several studies have described the possible side effects associated with prolonged use of SCS, such as the increased risk of sepsis, thromboembolism, diabetes, hypertension, glaucoma, osteopenia, and fractures. There is also evidence that suppression of cell-mediated immunity by SCS can lead to recurrent viral infections, pneumonia, and atypical bacterial infections such as tuberculosis [58, 59].

In our real-life experience, biologic therapy, administered according to AIFA guidelines, has proven efficacy in uncontrolled severe CRSwNP. We observed a clinical improvement, with a reduction in nasal polyposis as measured by NPS and an improvement in symptoms and their impact on QoL, especially in the olfaction, as demonstrated at SNOT-22, VAS, and SSIT-16. These results are in line with recent real-life studies and confirm the efficacy and safety of dupilumab in the treatment of severe CRSwNP [60, 61, 62].

In our experience, most patients presented a transient increase in blood eosinophils with spontaneous resolution. Many studies about dupilumab have described transient increases in eosinophil counts. Usually, such an increase occurs in the first few weeks of therapy and is followed by a subsequent return to baseline or even lower value by the end of the treatment period. Even though these increases are typically transient, clinicians should carefully monitor all patients [31]. In our cohort, we describe a case of irritative dermatitis related to an increase in eosinophils. Nitro et al. [61] reported a similar effect attributed to the biologic.

We also report a case of guttate psoriasis. Regarding dupilumab-related skin effects, Chromy et al. [63] described that dupilumab's blockade of IL4R*α* may lead to the conversion of the inflammatory cascade from Th2 to Th1 or Th17. Since psoriasis is a typical Th1-/Th17-mediated skin disorder, this switch to a Th1/Th17 phenotype may activate psoriasis-specific inflammatory cytokines and, thus, the disease.

Except for these cases, most reported side effects have been mild and transient, such as joint pain, irritation at the injection site, headache, and eye dryness. This finding aligns with the literature, according to which the most common adverse events are nasopharyngitis, injection site reactions, headache, asthenia, arthralgia, and conjunctivitis [64].

There were no clinically significant changes that could be attributed to the biologic in the other routine blood parameters evaluated, in line with literature concerning the use of dupilumab in the AD management [29].

In addition to the blood count, we evaluated the patients' immune status by assaying total IgE, IgG, and IgA. Dupilumab, by inhibiting IL-4 and IL-13, both involved in IgE synthesis, indirectly leads to a reduction in IgE levels, as confirmed in our results and already shown in other studies [65]. IgG is the most abundant class of immunoglobulins in serum, accounting for more than 80% of total serum Ig. There are four subclasses of IgG: IgG1, IgG2, IgG3, and IgG4 [66]. In our experience, we have seen a reduction in IgG levels during therapy. Otani et al. [67] also described the impact of dupilumab on IgG, showing how it specifically reduces IgG4 levels. Therefore, this study proposes biologic as a novel steroid-sparing treatment for IgG4-related disease (IgG4-RD), a rare fibroinflammatory, multisystemic condition. The use of dupilumab in this disease results from its effect of inhibiting both IL-4, which causes isotype switching from IgM to IgG4 and IL-13, which is involved in fibrosis [67]. Moreover, a recent update on IL-4 and IL-13 highlighted the role of IL-4 as a driver in Ig class switching to IgG1 and IgE. IL-13, on the other hand, is an effector cytokine that regulates mucus secretion and smooth muscle cell contraction in the airway epithelium [68]. Regardless of subclasses, several authors have underlined that Th2 cells produce IL-4, IL-5, and IL-13 in response to allergens or helminth antigens, thus promoting the production of all Ig classes [69].

Finally, we evaluated the impact on IgA, the second most abundant isotype in the serum after IgG, taking part in several protective functions. In addition, IgA plays a pivotal role in mucosal homeostasis in respiratory, gastrointestinal, and genitourinary tracts, functioning as the dominant antibody isotype in the mucosal immune system under the form of secretory IgA. Many studies have investigated IgA production in patients with CRS. At the serum level, all papers state that there are no significant differences between patients with CRS and controls [70]. Also, in our case series, patients with CRSwNP had a mean IgA value in the normal range, and dupilumab caused a reduction in this, remaining within the range. Serum IgA decreased as a possible result of Th2 cytokines inhibition by dupilumab. In fact, it has been described in literature that both IL-4 and IL-13 are required for IgA production [71]. Regarding the role of IL-4 in IgA production, recent studies report that this interleukin, combined with transforming growth factor-*β*1 and other cytokines, participates in IgA class switching [72]. Furthermore, Cerutti et al. [73] stated that IL-4, together with CD40 ligand, IL-10, and IL-6 are necessary to trigger switching to IgG, IgA, and IgE. This finding is in line with our data, in which biological therapy, by inhibiting IL-4 as well, caused a reduction in IgG, IgA, and IgE.

To the very best of our knowledge, this is the first study to evaluate the impact of dupilumab on several blood parameters in patients receiving treatment for CRswNP.

Biologic therapy has demonstrated broad efficacy in the management of patients with CRSwNP with [74, 75] and without asthma [76]. Our study confirmed the clinical improvement of dupilumab on nasal polyposis on symptoms (in particular the olfaction) and QoL. Regarding blood parameters to monitor in our series, only eosinophil count revealed a transient increase, which spontaneously resolved in almost all cases. Further studies are needed to confirm our results and to understand the underlying immunological mechanisms.

## Figures and Tables

**Table 1:** Patients' features.

Feature	Result
M/F	77 (59.3%)/53 (40.7%)
Mean age	56.8 ys (20–90)
Mean BMI	24.5 (19.1–38.7)
Smoke	30.7%
Asthma	54.5%
Allergy	66.9%
NSAIDs intolerance	14.6%
GERD	22%
Previous surgical treatment	83.2%
Single FESS procedure	42.4%
Two or more FESS procedures	40.8%

NSAIDs intolerance, nonsteroidal anti-inflammatory drugs intolerance; GERD, gastroesophageal reflux disease; ys, years; FESS, functional endoscopic sinus surgery. Data are reported as mean (range) or percentage.

**Table 2:** Changes after baseline.

		T0	T1	T2	T3
NPS		4.7 (1.7)	1.6 (1.5) ^*∗*^ ^*∗*^	0.9 (1.1) ^*∗*^	0.4 (0.6) ^*∗*^
					
SNOT-22		51.6 (20.2)	21.8 (15.6) ^*∗*^	17.7 (14.0) ^*∗*^	14.8 (12.3) ^*∗*^
VAS		35.4 (8.8)	13.8 (9.1) ^*∗*^	9.9 (8.4) ^*∗*^	8.2 (6.9) ^*∗*^
Olfaction	Anosmic	62.6%	11.4%	6.9%	0%
	Hyposmic	23.7%	24.5%	27.6%	6.7%
	Normosmic	13.7%	64.1%	65.5%	93.3%
Lymphocytes	cell × 10^9^/L	2.3 (0.6)	2.5 (0.7)	2.3 (0.8)	2.2 (0.6)
Neutrophils	cell × 10^9^/L	3.7 (0.9)	3.8 (0.9)	3.9 (0.8)	3.9 (0.8)
Basophils	cell × 10^9^/L	0.04 (0.0)	0.06 (0.0)	0.06 (0.0)	0.06 (0.0)
Eosinophils	cell × 10^9^/L	0.5 (0.4)	0.7 (0.5)	0.6 (0.5)	0.5 (0.5)
Total IgE	UI/ml	193.8 (165.6)	69 (57.8) ^*∗*^ ^*∗*^	59.1 (89.4) ^*∗*^ ^*∗*^	28.9 (25.8) ^*∗*^ ^*∗*^ ^*∗*^
IgG	mg/dl	1106.1 (226.7)	1,140.0 (292.9)	1,157.7 (174.9)	1,020.2 (51.8)
IgA	mg/dl	237.6 (102.9)	224.2 (120.4)	269.2 (191.4)	162.1 (49.7)

NPS, nasal polyp score; SNOT-22, Sinonasal Outcome Test-22 items; VAS, visual analog scale;  ^*∗*^*p* < 0.001;  ^*∗*^ ^*∗*^*p*=0.001. Values are reported as a mean (SD) or percentage.

## Data Availability

Data are available from the corresponding author on reasonable request.

## Abbreviations

AD: Atopic dermatitis
AIFA: Agenzia Italiana del farmaco
CRSwNP: Chronic rhinosinusitis with nasal polyps
ESS: Endoscopic sinus surgery
FESS: Functional endoscopic sinus surgery
GERD: Gastroesophageal reflux disease
Ig: Immunoglobulin
INCS: Intranasal corticosteroids
Mabs: Monoclonal antibodies
MCID: Minimal clinical important difference
NPS: Nasal polyp score
NSAIDs: Nonsteroidal anti-inflammatory drugs
QoL: Quality of life
SCS: Systemic corticosteroids
SNOT-22: Sinonasal Outcome Test-22 items
SSIT-16: Sniffin' Sticks-16 Identification Test
VAS: Visual analog scale.
